# Dexmedetomidine nasal spray to treat high-activity postoperative delirium: a case report

**DOI:** 10.3389/fmed.2025.1600737

**Published:** 2025-10-01

**Authors:** Yujing Liu, Lin Chen, Qian Liu, Peng Li

**Affiliations:** ^1^Department of Anesthesiology, Affiliated Hospital of Southwest Medical University, Luzhou, China; ^2^Department of Anesthesiology, Sichuan Provincial People's Hospital, School of Medicine, University of Electronic Science and Technology of China, Chengdu, China; ^3^Department of Anesthesiology, Wenjiang Hospital of Sichuan Provincial People’s Hospital (Chengdu Wenjiang District People’s Hospital), Chengdu, China

**Keywords:** dexmedetomidine, nasal spray, postoperative delirium, hyperactive delirium, non-intravenous sedation

## Abstract

High-activity postoperative delirium (POD) represents a prevalent neuropsychiatric complication during the perioperative period. Current clinical research predominantly focuses on preventive strategies, while effective pharmacological interventions remain limited, particularly for acute high-active subtypes. This case report demonstrates the successful use of dexmedetomidine nasal spray in managing acute high-active POD. The dexmedetomidine exerts its therapeutic effects through selective modulation of the locus coeruleus-noradrenergic pathway, providing physiological sedation resembling natural sleep architecture while preserving respiratory stability. It also exerts analgesic and anti-inflammatory effects. The nasal spray facilitated precise dosing with rapid symptom resolution and maintained hemodynamic stability. This novel approach offers a safe and effective way to manage acute high-active POD.

## Introduction

Postoperative delirium is a relatively frequent complication that occurs after surgery and presents as acute confusion involving disturbed consciousness, decreased concentration, and altered cognitive functioning (1). It increases the risk of adverse events, including death. Risk factors of postoperative delirium include age, history of delirium or preoperative comorbidities, emergency surgery, duration of surgery, use of anesthesia or psychotropic medication, and pain (2, 3).

Perioperative intravenous dexmedetomidine has been shown to reduce risk of postoperative delirium (4, 5), but such medication is less appropriate for treating postoperative delirium once it occurs because its inconvenience and the potential for overdose, which can cause transient hyper- or hypotension as well as bradycardia Potentially better for this purpose may be dexmedetomidine nasal spray, which is convenient to administer in well-controlled doses and shows high bioavailability (6). Here we present the case of a patient whose postoperative delirium in the post-anesthesia care unit was effectively treated using dexmedetomidine nasal spray. Prior studies have primarily addressed preventive measures for postoperative delirium; this clinical case complements existing evidence by detailing successful therapeutic interventions in a postoperative patient.

## Case report

A 39-year-old Chinese man, 167 cm tall and weighing 82.5 kg, was admitted to our hospital 2 h after suffering a car accident. No history of hypertension, diabetes mellitus, coronary heart disease, or other chronic conditions, and no family history of hereditary disorders. No smoking history, occasional alcohol consumption (≤2 drinks/week). No psychiatric history and no substance abuse. Upon admission, his myoglobin level was 302.15 ng/mL; white blood cell count, 12.99 × 10^9^/L; C-reactive protein, 21 mg/mL; and high-sensitivity C-reactive protein, >10 mg/mL. Computed tomography of the chest indicated multiple rib fractures, trace pneumoperitoneum, and some exudate foci in the left thoracic cavity. Cranial CT showed no significant abnormalities, and there were no signs and symptoms of elevated intracranial pressure, such as headache, nausea, and vomiting during hospitalization. Other tests were unremarkable. The patient was diagnosed preoperatively with bilateral rib fractures, traumatic pneumothorax, as well as pulmonary and chest wall contusion.

The patient underwent emergency surgery under general anesthesia for thoracoscopic lung repair, rib fracture osteotomy, and internal fixation. On the day of surgery, he was admitted to the operating room in a clear state of mind, without disordered consciousness or abnormal pathological reflexes. Throughout surgery, routine monitoring of SPO_2_, electrocardiography, PETCO_2,_ blood pressure, pulse, and breathing rate showed nothing unremarkable. General anesthesia was induced through sequential injection of dexamethasone sodium phosphate (10 mg), penehyclidine hydrochloride (0.5 mg), midazolam (2 mg), sufentanil (30 μg), ciprofol (32 mg), and rocuronium bromide (50 mg). The patient received assisted ventilation for 3 min, after which a left-sided bronchial tube (no. 35) was placed under the guidance of a visual laryngoscope and fiberoptic bronchoscope. Anesthesia was maintained during surgery through intravenous injection of sevoflurane (1–2%), remifentanil (0.1 mg/kg/h), and propofol (3 mg/kg/h). The surgery lasted 145 min, during which 50 mL of blood was lost, urine output was 100 mL, and 1,000 mL of crystalloid solution was transfused.

By 9 min after surgery, the patient was awake and extubated. Intravenous analgesic pump containing butorphanol tartrate (12 mg), tropisetron (10 mg), and flurbiprofen ester (300 mg) was administrated, then transferred to the post-anesthesia care unit. By 20 min after arrival, the patient’s pulse and blood pressure had increased, and he had transitioned from lethargy to severe agitation, confusion, allophasis and disorientation, meeting the diagnostic criteria of delirium according to the Confusion Assessment Method scale. He was given midazolam (2 mg) intravenously, which led him to fall asleep quietly. The patient showed glossoptosis and his oxygen saturation decreased to 88%, which was improved through placement of a high-flow oxygen mask. By 10 min after receiving midazolam, the patient once again experienced delirium symptoms, which did not improve during 30 min. He was given dexmedetomidine nasal spray (50 μg, Sichuan Purity Pharmaceutical, Chengdu, China), and 20 min later, he fell asleep quietly without glossoptosis or abnormal changes in pulse or oxygen saturation. It was similar to natural sleep.

**Figure 1:**
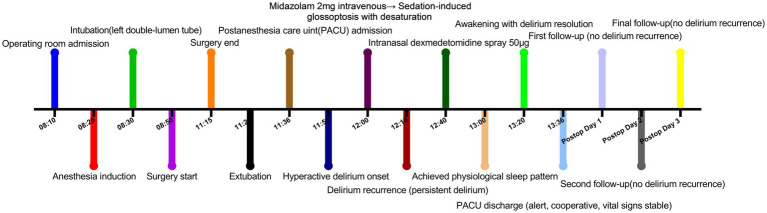
Timeline of perioperative events.

After the patient had slept for 40 min in this way, he was awakened and showed clear consciousness and normal tangential responses, sense of orientation, pulse, blood pressure, and oxygen saturation. The patient expressed satisfaction with his care and was surprised at how quickly the nasal spray relieved his agitation. He was escorted back to the ward. No recurrence of delirium was observed at 1, 3 or 7 days after surgery (Figure 1).

## Discussion

Here we describe what appears to be the first report that dexmedetomidine nasal spray can be effective against postoperative delirium after it occurs. This case is an important addition to the literature, which has focused on perioperative prevention of postoperative delirium, rather than on the treatment of delirium after it has occurred. That literature has established that perioperative intravenous administration of dexmedetomidine or benzodiazepines (7, 8), or perioperative nasal administration of dexmedetomidine (9, 10), can reduce the incidence of postoperative delirium.

When it occurs, postoperative delirium is typically treated through the correction of hydroelectrolytic imbalances and the implementation of targeted analgesic management alongside treatment of underlying etiologies or using antipsychotic drugs, benzodiazepines or dexmedetomidine. Our patient presented with so-called “high-activity” delirium involving agitation, hallucinations, and gibberish. Although intravenous dexmedetomidine may be equally effective in treating postoperative delirium, there is no doubt that intranasal dexmedetomidine would be more convenient and rapid. Patients with postoperative delirium often pull out their intravenous catheters. Dexmedetomidine nasal spray provides effective sedation without requiring intravenous access, facilitating subsequent procedures. Our clinical experience is consistent with the idea that nasal formulations of dexmedetomidine exert sedative and anxiolytic effects similar to those of intravenous formulations.

In contrast to nasal dexmedetomidine, midazolam proved ineffective for resolving our patient’s delirium. Several studies suggest that benzodiazepines may contribute to postoperative delirium, especially in elderly patients, while dexmedetomidine does not appear to be significantly associated with postoperative delirium (7). This difference may reflect different mechanisms of action. Midazolam inhibits neuronal excitation through activation of the GABA receptors to induce sedation; However, excessive inhibition may contribute to emergence agitation. Additionally, its moderate central anticholinergic activity reduces acetylcholine release, a mechanism implicated in postoperative delirium pathogenesis. Dexmedetomidine exerts its effects primarily through *α*_2_ -adrenoceptor agonism. By selectively activating presynaptic α₂-adrenoceptors in the locus coeruleus (LC), it suppresses norepinephrine release, thereby disinhibiting GABAergic neurons in the ventrolateral preoptic nucleus (VLPO) to promote physiological non-rapid eye movement (NREM) sleep with preserved respiratory function (11). This LC suppression also exhibits anti-sympathetic effects, reducing *β*₂-adrenoceptor-mediated pro-inflammatory cytokine production (TNF-*α*, IL-6). This action concurrently attenuates anxiety, agitation, and sleep disturbances triggered by surgical stress and pain. Additionally, direct α₂-receptor agonism on microglia inhibits NF-κB/STAT3 signaling to limit neuroinflammation. Dexmedetomidine modulates neuroinflammation by inhibiting pro-inflammatory cytokine release (e.g., IL-6, IL-1β, TNF-*α*) from microglia and astrocytes (12). These combined mechanisms—sleep regulation, neuroinflammatory control, intrinsic analgesic properties, and hemodynamic stabilization—collectively reduce the risk of postoperative delirium.

## Data Availability

The original contributions presented in the study are included in the article/supplementary material, further inquiries can be directed to the corresponding author.
